# Prevalence of and factors associated with daily smoking among Inner Mongolia medical students in China: a cross-sectional questionnaire survey

**DOI:** 10.1186/1747-597X-7-20

**Published:** 2012-05-16

**Authors:** Jiang Bian, Maolin Du, Zhiyue Liu, Yancun Fan, Yuki Eshita, Juan Sun

**Affiliations:** 1 Inner Mongolia Medical College, Hohhot, Inner Mongolia Minority Autonomous Region, China; 2 Oita University, Faculty of Medicine, Oita, Japan

**Keywords:** Daily smoking, Behavior, Medical students, Prevalence

## Abstract

**Background:**

To date, no study on smoking behavior of medical students in Inner Mongolia has been reported. The aim of the present study was to determine the 1-month prevalence of and factors associated with daily smoking among medical students in Inner Mongolia of China, to assist interventions designed to reduce the smoking behavior of medical college students in this region.

**Methods:**

During December 2010 and January 2011 a cross-sectional survey was conducted among medical students at the Inner Mongolia Medical College using a self-administered questionnaire. The questionnaire consisted of three sections: students’ basic information, attitude on smoking behavior, and smoking status of the student daily smokers. Students who smoked every day in the last 30 days were regarded as daily smokers. Factors associated with smoking were identified using binary logistic regression analysis.

**Results:**

A total of 6044 valid surveys were returned. The overall prevalence of daily smoking was 9.8% while the prevalence of daily smoking among males and females were 29.4% and 1.7%, respectively. Males in the Faculty of Medicine Information Management had the highest daily smoking rate (48.9%). Logistic regression models found that the main factors associated with daily smoking among male medical students were highest year of study (OR = 3.62; CI: 1.18–11.05); attitude towards smoking behavior *Do not care about people smoking around you* (OR = 2.75; CI: 2.08–3.64); and *Smoking is harmful to their health* (OR = 4.40; CI: 2.21–8.75). The main factor associated with daily smoking among female medical students was attitude towards smoking behavior *Eliminate smoking on campus* (OR = 0.11; CI: 0.06–0.23). Both for male and female medical students, there was no association between ethnicity and cigarette daily smoking. In regard to smoking status, more than 60% of daily smokers began smoking in high school, 61.3% smoked less than 5 cigarettes per day, 62.9% of the daily smokers’ families opposed their smoking behavior, and after an hour of not smoking 74.6% daily smokers did not feel uncomfortable.

**Conclusions:**

Antismoking education should be further promoted in Inner Mongolia medical students, with consideration given to the factors associated with daily smoking behavior found in the present study.

## Background

The World Health Organization (WHO) reported that an estimated 1.3 billion people in the world smoke [1] and that 47.5% of men smoke compared to 10.3% of women [2]. In his book review for *Cigarette Century*, Hall remarked that “Since the release of the Surgeon General’s Report on Smoking and Health in 1964, public health workers of worldwide have emphasized cigarette smoking as a health hazard of major importance” [3]. In recent years, as a growing economic power with the highest population of any country, China plays a substantial role in global public health [4] but it has also been noted that tobacco smoking is an important threat to public health in China [5]. Moreover, China is the world’s largest producer as well as consumer of tobacco, accounting for 37.5% of the global production and 38.8% of the global consumption [6]. The World Health Organization has estimated that there are 320 million smokers in China [7] and that tobacco-related diseases currently kill 1 million Chinese smokers each year [7]. If current smoking rates continue, smoking-attributable deaths in China are projected to rise to 2.2 million by the year 2020 [8]. Thus, the success of global tobacco control relies in large part upon the reduction of smoking in China.

It has been reported that smoking-cessation guidance and intervention by physicians have a significant effect on patients’ smoking behavior [9], and medical professionals can reduce smoking prevalence in society by offering smoking cessation advice to patients [10]. In 1999, the WHO took the position that physicians, as role models of healthy living, should not smoke and not overlook smoking in their patients [11]. More importantly, physicians are expected to play an important role in the campaign against smoking, which means not only giving advice to their patients but setting an example for them. Consequently, as medical students become future physicians, it is also important to determine the smoking status of medical students because it has been shown that among physicians, smoking status usually affects to what degree anti-smoking advice is provided [12]. According to the study of the Global Health Professions Student Survey (GHPSS) in 2011, tobacco control efforts need to discourage tobacco use among health professionals, especially medical students [13], and increase the extent of teaching on tobacco in medical schools over the next 10 years worldwide [14]. In addition, due to the need for future physicians to be well educated about tobacco control and smoking cessation, education at medical school might be the optimal time to introduce smoking cessation teachings [15]. Thus, medical schools have a crucial part to play in educating medical students about tobacco [16].

Inner Mongolia is a region which inhabited by Mongolian ethnic minority (one of five Minority ethnic autonomous region in China). Because there has been no study on smoking behavior for medical students in Inner Mongolia, the current survey was conducted among medical students of the Inner Mongolia Medical College with the aim of documenting the rate of daily smokers, their attitudes towards smoking behavior, and the smoking status of student daily smokers. In addition, our intent was to determine what factors are related to the daily smoking behavior of students. A longer-term goal was to use the survey results to assist with design of interventions to change smoking behavior of medical college students.

## Methods

### Setting and target population

A cross-sectional survey was conducted among medical students at the Inner Mongolia Medical College of China who resided on campus at the time of the survey. The survey focused on medical students and employed a self-administered questionnaire.

At the time of the survey, a total of 6677 students were present at the Inner Mongolia Medical College campus with nearly 3 times more female than male students. The students belonged to the faculties of Clinical Medical, Public Health Administration, Medicine Information Management, Medicine, Traditional Chinese Medicine, Mongolian Medicine, and others. The length of undergraduate education for Public Health Administration, Medicine Information Management, and Medicine is 4 years, while the length of undergraduate education for other faculties is 5 years. Students of the Public Health Administration and Medicine Information Management faculties have the same medical curriculum as the Clinical Medical faculty in the first 3 years with specialized curricula concerning their field in the last years. Clinical Medical, Traditional Chinese Medicine, and Mongolian Medicine students also have internships in various hospitals for their last 2 years, which is off campus. Consequently, for these faculties we only surveyed students in years 1–3. For students belonging to other faculties, in year 5 most are off campus taking internships. At the time of survey there were only 86 students taking two classes.

In terms of the Mongolian ethnic minority, we divided ethnicity into 3 categories: Han, Mongolian, and other. Participants’ ethnicity information came from their basic information contained in the school database.

### Smoking behavior

Initially, our intention was to categorize smokers according to the definition of the Centers for Disease Control and Prevention: current smokers include daily smokers (people who reported having smoked every day in the last 30 days), occasional smokers (people having smoked, but not every day), non-smoker, which includes former smokers (people who had smoked at least 100 cigarettes in their lifetime), and never-smoker (who had never tried or experimented with tobacco smoking) [17]. However, while using a pre-survey to determine which questions were appropriate for our students (see Questionnaires and measures section), we discovered that the terms occasional smoking behavior and former smoker were not well understood, for example, an occasional smoker might have only smoked one or less than one cigarette in their memory and been persuaded to try to smoke once by their smoking friends. In addition, many students were not sure whether they had smoked more than 100 or less than 100 cigarettes; most students reported only smoking one or less than one cigarette. We found a large proportion of the students in these situations, and according to the definition of occasional smokers or former smoker, it was difficult to determine how these students should be classified. Because of these issues, we decided to define smokers as daily smokers using the daily smoker definition of the Centers for Disease Control and Prevention and classify all other students as non-smokers, which means that estimates would be a 1-month prevalence for daily smoking only.

### Data collection procedure

All students in the Inner Mongolia Medical College campus were invited to participate in our survey with the exception of those who were absent during the survey period. To motivate students to take part in the survey, it was decided that this should be part of the students’ health education curriculum, with a corresponding credit at the end of the semester evaluation if students completed the questionnaire. Students completed the survey in the classroom. Investigators distributed questionnaires to each participant and asked the students to voluntarily complete the questionnaires in the classroom after study investigators explained the purpose of the study. Privacy regarding personal and enrollment data was clearly stated in the questionnaire. Throughout the course of the investigation, survey investigators were present to answer any participant questions regarding the questionnaire. Participants returned the completed self-administered questionnaire to the investigators some of whom checked the questionnaire while others counted the number of questionnaires. Finally, investigators put questionnaires in a sealed envelope.

We randomly sampled 100 students after the initial survey and asked them to complete the survey again, and compared the results of the two surveys to validate the survey questionnaire. We calculated the “test-retest” reliability, which refers to the measure of a test or survey consistency using the Kappa statistic for each item. Test-retest reliability is measured by administering a test survey at 2 different points in time, expressed as the proportion of a random sample of pairs that are concordant for a trait of interest [18].

### Questionnaire and measures

We developed our questionnaire from the pre-survey and used this questionnaire in our formal survey. The language of the questionnaire was Chinese. We collected questions from the relevant literature and considered whether these questions were appropriate for our students, and then tested whether the questions could be well understood by the students using a pre-survey. The final selection included such questions as *Smoking is a sign of civilization*[19]*, Smoking is harmful to one’s health*[20], and *the State should take measures to stop smoking*[21]. Our questionnaire consisted of three sections. The first section contained questions for students’ basic information, including gender, ethnicity, year of study, monthly expenses, residence, and faculty. The second section concerned the attitude regarding smoking behavior and included 9 questions. Because the pre-survey demonstrated that some students had difficulty using scales taken from the literature, we asked questions that needed yes or no answers. The third section was only answered by daily smokers, and contained 8 questions about the smoking status such as number of cigarettes smoked per day, whether their parents were smokers, and the number of smoking friends.

### Statistical analysis

We determined daily smoking prevalence distributed by gender in relation to various items, including ethnicity, year of study, monthly expenses, and faculty in the survey. Binary logistic regression analysis was used to ascertain factors associated with daily smoking. This technique is used for analyses with a dependent variable that has 2 categories (in this case, daily smoker and non-smoker). Independent variables included in the model were ethnicity (Han ethnicity as the reference group), year of study (year of study 1 as the reference group), monthly expenses (monthly expenses <300 yuan as the reference group), residence (city as the reference group), faculty (Clinical Medicine as the reference group), and the attitude of the participants to the smoking behavior (“No” as the reference group). We used binary logistic regression models to adjust for possible confounding influences between the independent variables on the dependent variable in each model. In all models, ORs >1.0 designated increased smoking risk and ORs <1.0 indicated protective factors.

Year of study was expressed as the mean age and standard deviation (SD). Statistical comparisons were made using one-way ANOVA with a post-hoc test (Student-Newman-Keuls) to evaluate differences in each year of study. *p* values less than 0.05 were considered statistically significant.

Quantitative data were recorded using EpiDate (EpiData Association, Denmark; v3.1) and then transferred into SPSS (SPSS, Inc., Chicago, IL, USA, v13.0). All statistical analyses were performed using SPSS for Windows v13.0, with a significance level of P < 0.05.

### Ethical approval

Ethical Approval to conduct the study in which consent was needed from all study participants was obtained from the Ethical Committee of Inner Mongolia.

## Results

### Participant characteristics

A total of 6047 students completed the questionnaire with 3 students failing to include basic information because the 3 students were informal students of the Inner Mongolia Medical College. Of the 6044 students for whom complete data was available, 1775 were male (29.4%) and 4269 were female (70.6%). The test-retest reliability was 96. 4%. The range of the Kappa index was 6.03 to 8.03, with a mean of 7.13.

The mean age of the participants ranged from 20.16 years in the first study year to 24.02 years in the fifth study year (*p* < 0.001), showing a linear increase in students mean age from first to fifth study year by one year. Consequently, physical age could be reflected by year of study and we used the year of study throughout our study and in our analysis.

### Prevalence of smoking

The prevalence of daily smoking in relation to each survey item by gender is shown in Table 1. The overall daily smoking prevalence among Inner Mongolia medical students was 9.8% and the prevalence of daily smoking among males was significantly higher than among female medical students (29.4% vs. 1.7%) and nearly 3 times higher among students whose monthly expenses were >1000 yuan compared to students whose monthly expenses were <300 yuan. The faculty of Public Health Administration and Medicine Information Management had the highest rate of smokers for both male (43.1%) and female medical students (2.6%).

**Table 1:** Prevalence of daily smoking among Inner Mongolia Medical College by gender and in relation to various parameters

	**Male**		**Female**		**Total**	
	**n**	**%**	**n**	**%**	**n**	**%**
**Ethnicity (N = 6044)**						
Han ethnicity	1285	28.1%	3033	1.6%	4318	9.5%
Mongolian ethnicity	387	33.6%	1018	1.8%	1405	10.5%
Other ethnicity	103	29.1%	218	1.8%	321	10.6%
**Years of study (N = 6044)**						
1	611	25.2%	1862	1.8%	2473	7.6%
2	608	28.3%	1319	1.6%	1927	10.0%
3	435	32.4%	838	1.3%	1273	11.9%
4	95	43.2%	190	1.6%	285	15.4%
5	26	50.0%	60	5.0%	86	18.6%
**Monthly expenses (N = 6025)**						
<300	69	24.6%	399	1.5%	468	4.9%
300–600	831	25.9%	2352	1.7%	3183	8.0%
600–1000	754	32.9%	1327	1.7%	2081	13.0%
>1000	117	34.2%	176	2.8%	293	15.4%
**Residence (N = 6022)**						
City	636	26.9%	1615	1.6%	2251	8.8%
Rural	988	30.1%	2354	1.7%	3342	10.1%
Pastoral	50	50.0%	145	1.4%	195	13.8%
Suburbs	94	28.7%	140	2.9%	234	13.2%
**Faculty (N = 6044)**						
Clinical Medicine	764	30.9%	1430	2.5%	2194	12.4%
Public Health Administration and Medicine Information Management	255	43.1%	547	2.6%	802	15.5%
Medicine	287	23.3%	662	0.5%	949	7.4%
Traditional Chinese Medicine and Mongolian Medicine	347	24.5%	677	1.6%	1024	9.4%
Other	122	18.9%	953	0.8%	1075	2.9%
**Total (N = 6044)**	1775	29.4%	4269	1.7%	6044	9.8%

### Smoking-related perceptions and attitude

Results presented in Table 2 include the number of participants answering Yes/No in relation to each question about smoking-related perceptions and attitude and frequency of daily smoking (%), which refers to the percentage of respondents in each category (Yes/No) who were also daily smokers. The smoking rate among the students who did not agree with the attitude that *smoking is one of the causes of air pollution* was over 10 times higher than whose agreed with this attitude (Table 2); it was also over 5 times higher for those who did not agree with the attitude *eliminate smoking in classroom* compared to those who agreed with this attitude.

**Table 2:** Various attitudes of the participants in relation to their smoking behavior

**Category**	**n**	**Frequency of Daily Smoking (%)**
Do not care about people smoking around you (N = 6041)		
No	4727	5.2
Yes	1314	26.6
Smoking is a sign of civilization (N = 6044)		
No	4050	9.6
Yes	1994	10.3
Smoking is one of the causes of air pollution (N = 6041)		
No	780	46.9
Yes	5261	4.3
Teachers should not smoke (N = 6044)		
No	1458	17.5
Yes	4586	7.4
State should take measures to stop smoking (N = 6043)		
No	1252	28.0
Yes	4791	5.1
Eliminate smoking on campus (N = 6043)		
No	1446	31.6
Yes	4597	3.0
Eliminate smoking in classroom (N = 6043)		
No	458	41.9
Yes	5585	7.2
Smoking is harmful to one’s health (N = 6044)		
No	237	13.1
Yes	5807	9.7
Smoking is harmful to the health of others (N = 6043)		
No	280	22.1
Yes	5763	9.2

### Factors associated with smoking

We included all factors in binary logistic regression models (Table 3). Male medical students in the first year of study were less likely to be daily smokers compared with medical students in years 2–5. Higher monthly expenses were also associated with daily smoking behavior. Male students in Public Health Administration and Medicine Information Management were more likely to be daily smokers compared with students in other faculties. Five of the nine attitudes of students toward smoking behavior showed statistical significance: (1) *Do not care about people smoking around you*; (2) *Smoking is one of the causes of air poll*ution; (3) *The State should take measures to stop sm*oking; (4) *Eliminate smoking o*n campus*;* and (5) S*moking is harmful to o*ne’s health (*p* < 0.05; Table 3). Male medical students who agreed with the attitude *Do not care if people smoke around you* (OR: 2.75) were more likely to smoke daily compared to those who did not agree with this statements.

**Table 3:** Results of logistic regression analysis of smoking by gender among medical students

**Category**	**Male**				**Female**			
	**Crude OR (95%CI)**	***P***	**Adjusted OR (95%CI)**	***P***	**Crude OR (95%CI)**	***P***	**Adjusted OR (95%CI)**	***P***
**Ethnicity**		0.115		0.940		0.953		0.862
Han ethnicity	1.00				1.00			
Mongolian ethnicity	1.30 (1.02–1.65)		1.04 (0.74–1.46)		1.07 (0.62–1.85)		0.86 (0.41–1.82)	
Other	1.05 (0.68–1.64)		0.93 (0.51–1.68)		1.12 (0.40–3.12)		1.23 (0.35–4.35)	
**Year of study**		<0.001		<0.001		0.337		0.182
1	1.00				1.00			
2	1.17 (0.91–1.51)		1.08 (0.76–1.53)		0.87 (0.50–1.51)		0.96 (0.47–1.95)	
3	1.42 (1.09–1.87)		1.65 (1.12–2.43)		0.72 (0.36–1.42)		0.52 (0.21–1.28)	
4	2.25 (1.44–3.52)		3.85 (2.06–7.20)		0.86 (0.26–2.84)		1.11 (0.20–6.22)	
5	2.97 (1.35–6.54)		3.62 (1.18–11.1)		2.83 (0.84–9.49)		3.94 (0.83–18.59)	
**Monthly expenses(yuan)**		0.010		0.024		0.689		0.580
<300	1.00				1.00			
300–600	1.07 (0.60–1.89)		1.06 (0.51–2.23)		1.10 (0.46–2.63)		1.81 (0.56–5.63)	
600–1000	1.50 (0.85–2.65)		1.57 (0.75–3.31)		1.10 (0.45–2.74)		2.12 (0.59–7.60)	
>1000	1.59 (0.82–3.10)		2.07 (0.85–5.03)		1.91 (0.58–6.36)		3.23 (0.59–17.60)	
**Residence**		0.008		0.013		0.740		0.740
City	1.00				1.00			
Rural	1.17 (0.94–1.46)		1.63 (1.18–2.26)		1.06 (0.64–1.74)		1.45 (0.71–2.98)	
Pastoral	2.72 (1.52–4.86)		2.32 (1.08–4.97)		0.86 (0.20–3.64)		0.87 (0.16–4.80)	
Suburbs	1.10 (0.68–1.77)		1.33 (0.67–2.62)		1.80 (0.62–5.23)		1.13 (0.26–4.90)	
**Faculty**		<0.001		0.016		0.003		0.006
Clinical Medicine	1.00				1.00			
Public Health Administration and Medicine Information Management	1.70 (1.27–2.27)		1.26 (0.84–1.88)		1.02 (0.54–1.90)		0.83 (0.36–1.94)	
Medicine	0.68 (0.50–0.93)		0.64 (0.42–0.98)		0.18 (0.05–0.57)		0.12 (0.03–0.48)	
Traditional Chinese Medicine and Mongolian Medicine	0.73 (0.54–0.97)		0.87 (0.59–1.28)		0.64 (0.32–1.26)		0.53 (0.21–1.34)	
Other	0.52 (0.32–0.84)		0.49 (0.26–0.93)		0.33 (0.15–0.71)		0.30 (0.11–0.78)	
**Do not care about people smoking around you**		<0.001		<0.001		<0.001		0.067
No	1.00				1.00			
Yes	4.88 (3.92–6.07)		2.75 (2.08–3.64)		5.85 (3.66–9.36)		1.81 (0.96–3.43)	
**Smoking is a sign of civilization**		0.126		0.766		0.641		0.914
No	1.00				1.00			
Yes	1.18 (0.95–1.47)		0.96 (0.71–1.29)		0.89 (0.54–1.47)		0.96 (0.48–1.92)	
**Smoking is one of the causes of air pollution**		<0.001		<0.001		<0.001		<0.001
No	1.00				1.00			
Yes	0.10 (0.08–0.13)		0.23 (0.16–0.31)		0.02 (0.01–0.04)		0.04 (0.02–0.08)	
**Teachers should not smoke**		<0.001		0.582		<0.001		0.350
No	1.00				1.00			
Yes	0.36 (0.29–0.45)		1.09 (0.80–1.50)		0.34 (0.21–0.54)		1.36 (0.72–2.58)	
**The State shall take measures to stop smoking**		<0.001		<0.001		<0.001		0.360
No	1.00				1.00			
Yes	0.18 (0.14–0.22)		0.53 (0.39–0.73)		0.10 (0.06–0.17)		0.72 (0.36–1.45)	
**Eliminate smoking on campus**		<0.001		<0.001		<0.001		<0.001
No	1.00				1.00			
Yes	0.09 (0.07–0.11)		0.18 (0.13–0.24)		0.05 (0.03–0.10)		0.11 (0.06–0.23)	
**Eliminate smoking in classroom**		<0.001		0.075		<0.001		0.043
No	1.00				1.00			
Yes	0.17 (0.13–0.23)		0.69 (0.46–1.04)		0.08 (0.05–0.13)		0.41 (0.18–0.97)	
**Smoking is harmful to one’s health**		0.967		<0.001		0.680		<0.001
No	1.00				1.00			
Yes	1.01 (0.64–1.59)		4.40 (2.21–8.75)		0.78 (0.24–2.52)		28.31 (4.67–172.58)	
**Smoking is harmful to the health of others**		0.005		0.216		<0.001		0.441
No	1.00				1.00			
Yes	0.59 (0.41–0.85)		1.46 (0.80–2.65)		0.22 (0.11–0.43)		0.62 (0.18–2.11)	

OR: Odds Ratio, CI: Confidence Interval.

*p* values less than 0.05 were considered statistically significant.

For female medical students, three of the nine attitudes of students toward smoking behavior showed statistical significance: (1) *Smoking is one of the causes of air poll*ution; (2) *Eliminate smoking on campus*; and (3) S*moking is harmful to one’s health* (*p* < 0.05; Table 3). With regard to the two attitudes (1) and (2), female medical students who answered yes were less likely to be a daily smoker.

However, both for male and female medical students, there was no association between ethnicity and cigarette daily smoking.

### The smoking status of daily smokers

For daily smokers it was established that 84.8% had begun smoking before college admission (Table 4). Over half of the daily smokers smoked less than five cigarettes per day and did not feel uncomfortable if they did not smoke for an hour. Also, over half of the families of daily smokers opposed their smoking behavior and 65.5% of the parents of daily smokers did not smoke. In addition, we observed that 62.7% of daily smokers had attempted to quit smoking—some as many as three times. More than 70% of daily smoking students had more than five smoking friends, and nearly half of the daily smokers had more than ten friends who smoked.

**Table 4:** Various characteristics of the daily smokers in Inner Mongolia Medical College

**Category**	**n**	**(n/N) (%)**
Number of cigarettes smoked per day (N = 582)		
<5	357	61.3
5–9	180	30.9
>10	45	7.7
Number of smoking friends (N = 590)		
<5	147	24.9
5–10	151	25.6
>10	292	49.5
Parent smoker (N = 589)		
Yes	203	34.5
No	386	65.5
Quit smoking number of attempts (N = 588)		
Never	219	37.2
1–2	213	36.2
>3	156	26.5
The first time smoking (N =591)		
University	90	15.2
High School	377	63.8
Junior high school	124	21.0
The attitude of your family on you smoking (N = 590)		
Opposed	371	62.9
Don’t Care	208	35.3
Approve	11	1.9
An hour not smoking (N = 590)		
No discomfort	440	74.6
Can endure	119	20.2
Intolerable	31	5.3
You feel after smoking (N = 586)		
Better	119	20.3
Worse	80	13.7
No change	387	66.0

## Discussion

Our study shows that the 1-month daily smoking prevalence among medical students is 9.8% (Table 1). This is considerably higher compared to the recently reported prevalence of daily smoking among medical students in Western countries; 3% in Finland [21], 4.41% in France [22], and 4% to 5% in Australia [23] but lower compared to other Asian developed countries, such as Japan (13.7%) [24] although the smoking prevalence of male students (29.4%, Table 1) is significantly higher than Japanese male medical students (18.1%) [24]. In contrast, lower rates of daily smoking for medical students have been reported in other areas of China: 3.9% for Tongji Medical College and 6.5% for the Medical College of Wuhan University [25].

Anti-smoking campaigns in the developed world were instituted in the mid 1980s [3] and considerable reductions in the prevalence of smoking have been achieved. For example, the prevalence of smoking among males in the 20 to 24 years of age group has seen a major decline from about 45 to 30% [26]. These are the same ages as the students of the Inner Mongolia Medical College. We note that male students in the faculty of Public Health Administration and Medicine Information Management had the highest daily smoking prevalence, similar to the 45% level previously mentioned. This level is likely to be higher as we only determined prevalence based on daily smoking.

Several findings in current study can be used to inform the development of effective health promotion programs to prevent smoking among medical college students. First, our study shows the prevalence of daily smoking among men was significantly higher than among women. These data not only confirm the gender difference in smoking rates reported in a number of recent studies [21,23,24,27-33] but also reveal a similar finding to that obtained in other Chinese areas [30,32]. However, there is concern that a bigger increase in daily smoking among female medical college students compared to male medical college students is taking place. Even though recent national data from comparable age and education groups are not available, the 1996 China National Tobacco Survey reported a smoking rate of 18.5% among male college students and 0.8% among female college students [34]. Compared with this 1996 survey, the daily smoking rates observed in our study are higher with increases of 59.8% and 126.7% for male and female students, respectively (again smoking prevalence rates are likely to be higher because we only determined prevalence based on daily smoking). Because of the substantial gender difference in smoking practice, school antismoking health promotion efforts need to consider gender-specific approaches and content in order to meet the specific needs of men and women in medical colleges. The results of our study also suggest that smoking prevention programs are important for females to maintain low smoking rates, while smoking cessation programs should strongly focus on males to elicit cessation.

Second, in contradiction to other studies [35], we did not find that smoking prevalence among different ethnic groups was different even though Inner Mongolia is considered one of China’s five minority areas. We speculate that the reason for this result could be because these ethnic groups have resided in the area a long time. This finding suggests that it is not necessary for a tobacco control policy directed at medical students to consider differences between Han and Mongolian ethnicities.

Third, our research results showed that male students who had higher monthly expenses were more likely to be daily smoker than those who had lower monthly expenses, similar to previous research results [20,24]. We speculate that the reason for this result could be that the cost of smoking for students who had low monthly expenses (<300 yuan) is a higher proportion compared to students with higher monthly expenses (>1000 yuan), and thus there may be an economic incentive for students with lower monthly expenses to give up smoking. With China’s economic growth, the increase of student living expenses is an inevitable trend and this problem will become more important in the future. Thus, school administrators should consider formulating corresponding policies of intervention and counseling in regard to the distribution of monthly living expenses of students to enable students to rationally allocate expenses over which they have control. However, while higher monthly expenses appear to be a risk factor in regard to male medical students’ smoking behavior, there could be other explanations in addition to economic-based ones that are masked to us, and this issue would need to be resolved before formulating policies.

Fourth, the prevalence of daily smoking showed a rising trend during year of study. Clearly higher year of study is a risk factor in regard to male medical students’ smoking behavior. Most surveys have shown an increasing incidence of smoking with higher age [22,24,29,32-34,36]. This may be the result of more and more male students taking up smoking (cohort effect) while an extremely small proportion have quit smoking in the same age group.

Finally, the prevalence of daily smoking for students studying in Public Health Administration and Medicine Information Management was higher than for other faculties. This situation should be addressed as students in these faculties are not only future public health managers, but may be involved in formulating tobacco control policies

Our findings indirectly confirm some previous research results in China. For example, social reinforcement, such as smoking among friends and receiving smoking-related gifts is strongly associated with cigarette smoking [37]. As a Chinese traditional concept, smoking is accepted as a catalyst of friendship and social activities, and is used to develop personal and business relationships [38]. In our study, there is some evidence to support these findings: 75.1% of daily smoking students have more than 5 smoking friends, and in particular, 49.5% daily smokers of the medical college have more than 10 friends who smoke (Table 4). Notably, our results indicate that factors of social reinforcement associated with smoking among medical students are no different from those found in the general population. In addition, we observed that more than 62.7% of medical students who were daily smokers had attempted to quit smoking—some as many as three times; however, they were still smoking (Table 4). Clearly, the cessation of smoking is likely to be more difficult when a student has many friends who smoke. Considering the situation of individual daily smokers, smoking cessation is not impossible because over half of the daily smokers smoked less than five cigarettes per day and did not feel uncomfortable if they did not smoke for an hour. Clearly these students may not have a very strong dependence on tobacco although most smokers begun smoking from high school. We think that family opposition attitude toward smoking behavior is one of the reasons for this result. In particular, some studies have concluded that parents’ negative attitudes toward smoking behavior [39] and parents who do not smoke [40] are two factors that protect adolescents from smoking. In our study, 62.9% of the parents of daily smokers were opposed their smoking behavior and 65.5% of the parents of daily smokers did not smoke (Table 4). Thus, family attitudes to some extent reduce the smoking behavior of students although they do not completely prohibit them from smoking.

Both male and female medical students who have attitudes of *smoking is one of the causes of air pollution* and *eliminate smoking on campus*, appear to be protective factors in regard to daily smoking behavior. Male medical students who *do not care if people smoke around them* are more likely to be daily smokers, but having an attitude of *the State should take measures to stop smoking* appears to be a protective factor in regard to smoking behavior (Table 3). Because medical students have medical knowledge and thus some understanding of the health hazards of smoking, they might have an important role to play in tobacco control programs. When it comes to countries and tobacco control, the phrase “while thinking globally, each acts locally” [3] means that country-specific programs and approaches are likely to be different. In China, this could take the form of medical students being given effective measures to change the attitude of students to smoking behavior rather than emphasis on being given information about the harmful health effects of tobacco smoking. In addition, school administrators should encourage them to actively participate in tobacco control training and education to improve attitudes toward smoking, to increase protective factors, and reduce the risk factors for smoking behavior.

The present study had some limitations. First, we underestimated the overall prevalence of smoking because we defined smokers as those who smoked daily. Second, we did not employ scales (e.g., Likert scales) in response to attitude questions. Third, some questions in the third section of the questionnaire should have been placed in the second section; the third section was only answered by daily smokers and not all participants. Last, validation of the survey could have been more comprehensive and this was not undertaken.

## Conclusions

A high 1-month daily smoking prevalence was demonstrated among medical students in the Inner Mongolia area of China. Our study’s findings could help health care professionals develop targeted tobacco control policies for the population of students in Inner Mongolia of China and ensure the policies are more rational, useful, and effective.

## Competing interests

The authors declare that they have no competing interests.

## Authors’ contributions

JS and JB conceived the study. JB performed the statistical analysis and wrote the draft of the manuscript. JS and MD participated in the design of the study. ZL, YF and YE contributed the concept and critically revised the manuscript. All authors approved the final manuscript.
